# Fortified blended flour supplements displace plain cereals in feeding of young children

**DOI:** 10.1111/mcn.13089

**Published:** 2020-09-29

**Authors:** Ilana R. Cliffer, William A. Masters, Beatrice L. Rogers

**Affiliations:** ^1^ Department of Food and Nutrition Policy and Programs Friedman School of Nutrition Science and Policy, Tufts University Boston Massachusetts USA

**Keywords:** Burkina Faso, complementary feeding, displacement, fortified food, infants and young children, substitution, supplementary feeding

## Abstract

Lipid‐based nutritional supplements (LNS) and fortified blended flours (FBF) are widely used to increase the nutrient density of children's diets and improve their health, but their effectiveness could be modified by displacement of other foods. We reanalysed data from a cost‐effectiveness trial comparing impacts on anthropometry of three FBFs (Corn Soy Blend Plus [CSB+], Corn Soy Whey Blend [CSWB], SuperCereal Plus [SC+]) and one LNS (Ready‐to‐use Supplementary Food [RUSF]) among infants aged 7–23 months in Burkina Faso. Using dietary diversity data from a single 24‐h recall period (*n* = 1,591 children, observed once over 18‐month study period), we fit logistic regression models to estimate differences in intake of each food group making up the infant and young child minimum dietary diversity score and linear models to test for differences in dietary diversity score among children in each supplement arm. We tested for differences in breastfeeding time using the subsample for which breastfeeding was observed (*n* = 176). Children who consumed one of the three FBFs had lower odds of consuming household grains, roots and tubers compared with the LNS consumers (odds ratios [ORs] = 0.35–0.47; 95% confidence intervals [CIs]: 0.20–1.05). Consumption of other foods, dietary diversity and breastfeeding did not differ significantly at the 5% significance level. FBFs displaced the household's own cereals more than LNS, with no difference in the child's consumption of other more nutrient‐rich family foods. Given limited stomach capacity and feeding time, providing fortified cereals may help improve children's overall diet quality in settings where children would otherwise be fed nutrient‐poor root crops or cereal grains.

**Key messages**

- Infants need higher nutrient density than the foods used for family diets in low‐income countries, due to small stomach size and rapid growth velocity.
- Foods that are premixed with fortificants can meet infant needs, but their effectiveness is modified by displacement of other food.
- We find that supplementation with a fortified flour for porridge leads to lower odds of also feeding plain cereal, compared with supplementation with a lipid‐based fortified nut butter.
- Fortified cereals can improve diet quality more effectively than other supplementation strategies, by displacing plain cereal without altering breastfeeding or other intake.

## 1 INTRODUCTION

### 1.1 Complementary feeding and growth

Growth faltering often occurs during complementary feeding around 6 to 20 months of age, when the child is transitioning from exclusive breastfeeding to the family diet (Dewey, 2001; Stephenson et al., 2017; Victora, De Onis, Hallal, Blössner, & Shrimpton, 2010). Complementary feeding brings exposure to infectious pathogens that cause disease and malabsorption of nutrients (Millward, 2017) and may provide insufficient quantity and variety of nutrient‐rich foods required for optimal growth as recommended by the Pan American Health Organization (PAHO) and the World Health Organization (WHO) (Kimmons et al., 2005; Stephenson et al., 2017; World Health Organization and Pan American Health Organization, 2003). Infants and young children must absorb high levels of nutrients to support their rapid potential growth, but they have small stomachs and typically eat small quantities of each food especially if they are still breastfeeding, so meeting their needs requires complementary foods that are higher in nutrient density than the family diet (Dewey, 2013). Inadequate nutrient intake as well as pathogen exposure contributes to stunting (low length/height‐for‐age) and wasting (low weight‐for‐length) that account for up to 21% of disability adjusted life‐years lost among children under 5 years (Black et al., 2008).

In West Africa, and Burkina Faso particularly, where the agro‐ecological and demographic characteristics of the region make it difficult to grow or trade a variety of crops, suboptimal complementary feeding is common (Atuobi‐Yeboah, Lartey, Colecraft, & Otoo, 2016; Issaka et al., 2015a; Krasevec, An, Kumapley, Bégin, & Frongillo, 2017; Sawadogo et al., 2010). Most complementary foods in Burkina Faso are composed of cereals (millet, sorghum, maize and rice), which are often combined with sugar and water to make a porridge (Sawadogo et al., 2010). This may provide sufficient dietary energy but lacks the protein, fats and micronutrients needed for child growth and development (Issaka et al., 2015b; Sawadogo et al., 2010). The high phytate content of such grain‐rich diets may also limit absorption of those micronutrients which are present, particularly iron (Dewey & Vitta, 2013), and the bulkiness of the available staples further limits the total quantity and diversity of foods consumed (Issaka et al., 2015b; Sawadogo et al., 2010).

### 1.2 Food supplementation

Supplementary feeding programs aim to improve complementary feeding by providing nutrient‐dense foods to children. They are an integral part of the recommended package of nutrition interventions to reduce stunting and wasting (Bhutta et al., 2013; De Pee et al., 2015). However, the effectiveness of supplementary feeding programs in treating and preventing undernutrition is unclear. Most studies have shown modest improvements in stunting, linear growth and wasting status after the provision of either lipid‐based or flour‐based fortified supplements (Christian et al., 2015; Cliffer et al., 2020; Dewey & Arimond, 2012; Fabiansen et al., 2016, 2017; Hess et al., 2015; Isanaka et al., 2009, 2010; Nackers et al., 2010; Nikiema et al., 2014; Stephenson et al., 2017; Stobaugh et al., 2016). Many also report declines in length‐for‐age z‐score in all study groups, including those who have received supplementation with various foods (Bisimwa et al., 2012; Christian et al., 2015; Cliffer et al., 2020; Dewey & Arimond, 2012; Huybregts et al., 2012).

### 1.3 Displacement

The net impact of supplementation programs on the nutrient‐density of the diet may be an important factor influencing the effectiveness of supplementary feeding programs. If supplements replace plain household cereals but not more nutrient‐dense family foods, the nutrient‐density of the diet would improve. If, however, supplements displace nutrient‐dense foods, diet quality could worsen.

A number of studies have looked at the impact of different types of supplementary foods on dietary diversity, energy intake and breastfeeding. Some have found supplementary foods to be important contributors to daily micronutrient needs that do not displace household foods (Campbell et al., 2018; Flax, Siega‐Riz, Reinhart, & Bentley, 2015; Ickes et al., 2015) and may actually increase the odds of achieving acceptable dietary diversity (Campbell et al., 2016). Others have investigated differential displacement of household foods by lipid‐based nutritional supplements (LNS) and fortified blended flours (FBFs) and found evidence suggesting that the LNS may be less likely to displace home complementary foods and breastfeeding than their flour‐based counterparts (Lin, Manary, Maleta, Briend, & Ashorn, 2008), resulting in overall higher energy intake among those given LNS (Maleta et al., 2004; Thakwalakwa et al., 2015).

With regard to breastfeeding, a number of studies, all but one of which measured breastfeeding using deuterated water (and one that measured using observed frequencies), showed no significant displacement of breastmilk by LNS (Campbell et al., 2016; Galpin et al., 2007; Kumwenda et al., 2014; Owino et al., 2007). However, others that measured breastmilk using test weighing corrected for losses found that breastmilk was displaced when infants were fed highly energy‐dense diets, which led to consumption of fewer total calories in one study (Bajaj, Dubey, Nagpal, Singh, & Sachdev, 2005), but did not decrease total energy intake in two additional studies (Islam et al., 2008; Islam, Peerson, Ahmed, Dewey, & Brown, 2006).

Most of the above studies looked at displacement in the context of treatment programs (vs. prevention) or were conducted in heavily controlled environments in which caregivers were monitored closely by research staff; we have found no studies that have assessed displacement in the context of a ‘true‐to‐life’ blanket supplementary feeding program aimed at preventing stunting and wasting. The conditions and motivations of the caregivers that decide which foods to give to children or how often to breastfeed may be different for those with healthy children in a prevention program than those with sick children in a treatment program. In addition, we are aware of no studies that have investigated which specific food groups get displaced by food supplementation. This question is important in understanding caregivers' feeding behaviours when different types of supplementary foods are provided.

We reanalysed data from a trial comparing the cost‐effectiveness of three FBFs and one LNS in the prevention of stunting and wasting to explore differential effects of these supplementary foods on displacement of breastfeeding or household complementary foods and investigate which specific food groups get displaced, if any. We hypothesized that because the LNS is a product that does not physically resemble ordinary household staple cereals such as millet or sorghum flours, it may be considered a novelty item, and will thus not displace these staples, but may displace household nutrient‐dense foods (vegetables, beans, peanuts) or breastmilk. The FBFs, however, bear a close physical resemblance to the most common ordinary household staple cereals in Burkina Faso, although in reality they are much more nutrient‐dense because they have enhanced micronutrient and macronutrient profiles. Supplementation with an FBF may lead caregivers to substitute the FBF for their normal household cereal grains, which would increase nutrient‐density, because the FBF contains extra micronutrient and macronutrient.

## 2 METHODS

### 2.1 Study design

Dietary diversity data from a single 24‐h recall and direct observations of breastfeeding time over a 4‐day period were collected from children 7–23 months during a supplementary feeding program cost‐effectiveness trial conducted in Sanmatenga Province, Burkina Faso. Details of the trial methods and results are described elsewhere (Cliffer et al., 2020). Briefly, the Sanmatenga Province was split into four geographic regions, and each of the regions (and all distribution sites therein) was randomly assigned to one of four foods, which were distributed to all pregnant and lactating mothers and children 6–23 months, regardless of nutritional status (i.e., blanket supplementary feeding). Children were enrolled into the main study on a rolling basis once they reached 6 months of age, until the desired sample size was achieved. Data for the present analysis were collected once children had been receiving the ration for at least 1 month, restricting the age range for this study to 7–23 months.

The primary objective of the original study was to compare monthly rations of ~500 kcal/day per enrolled child of each of the four foods: Corn Soy Blend Plus (CSB+) delivered with Fortified Vegetable Oil (FVO) (Milling, 2014), Corn Soy Whey Blend (CSWB) with FVO (Milling, 2014), SuperCereal Plus (SC+) (Challenge Dairy Products Inc., 2014) and Ready‐to‐Use Supplementary Food (RUSF) (Edesia, 2014). A comparison of the nutrient composition of the foods can be found in Table 1 of the main cost‐effectiveness results paper (Cliffer et al., 2020). Participants were instructed to prepare the three FBFs (CSB+ w/oil, CSWB w/oil and SC+) into a porridge for the child, using the FBF as they would their normal household cereals. The RUSF, which is an LNS, was to be served to the child directly from the packet. Our data on household use of these products show that the CSB+ w/oil and the CSWB w/oil were most often served to children as a porridge as instructed (98% of the time), but that the SC+, which has a sweeter taste and is a finer flour than CSWB or CSB+ (Saleh, 2013), was given to children to consume raw, directly from the package, 20% of the time. The distribution program staff communicated to the recipient caregivers that the foods were intended to be supplements for the child's diet, and not replacements for household foods.

**TABLE 1:** Specification of outcome variables

Variable	Specification
Dietary diversity score	Self‐report of consumption of the below food groups within the 24‐h period prior to the interview. Indicator is sum of each of the seven groups below (continuous score range: 0–7)
Grains, roots and tubers	Cereals: millet, sorghum, rice, maize, wheat, fonio
	Roots and tubers: cassava, yams, white potatoes, taro, fabirama, plantain
Legumes and nuts	Legumes: beans, groundnut, peas, chickpea, lentils, legumes, soy, etc.
	Peanuts
	Peanut butter
	Sesame
	Oil seed
Dairy	Milk (animal source, powdered), yogurt, cheese
Flesh foods	Liver
	Meat
	Giblets
	Insects
	Small fish
	Other fish
Eggs	Eggs
Vitamin A‐rich fruits and vegetables	Orange flesh sweet potato
	Dark leafy greens: sorrel, amarranth, spinach, baobob, etc.
	Dark red or orange vegetables: pumpkin, squash, carrots, red peppers
	Fruits rich in vitamin A: mango, dark red or orange papaya, dark orange melon, néré, etc.
Other fruits and vegetables	Other fruits: bananas, pineapple, tamarind, wild fruits
	Other veggies: fresh tomato, okra, zucchini, eggplant, onions, cabbage, cucumbers, lettuce, etc.
Ration consumption	Self‐report of consumption of FBFs (CSB+ with oil, CSWB with oil, SC+) or LNS (RUSF) in the 24‐h prior to the interview
Breastfeeding	Breastfeeding time on Day 4 of 4‐day, 12‐h (6:00–18:00) observation (day prior to 24‐h food recall)

*Note*: Insects are not included in the World Health Organization (WHO) indicator for dietary diversity but were deemed appropriate by nutrition experts in Burkina Faso.

Abbreviations: CSB+, Corn Soy Blend Plus; CSWB, Corn Soy Whey Blend; FBF, fortified blended flour; LNS, lipid‐based nutritional supplements; RUSF, Ready‐to‐use Supplementary Food; SC+, SuperCereal Plus.

### 2.2 Data collection

Data were collected between July 2014 and December 2016; in‐depth interviews were conducted with caregivers of a subset of children enrolled in the main supplementary feeding study (*n* = 1,591) and 4‐day in‐home observations on a subset of those interviewed (*n* = 176). Random numbers were generated to select caregivers from the larger pool of participants. During interviews, in addition to answering questions about perceptions and use of the study foods, caregivers were asked to report everything their child consumed during the previous 24‐h period (starting from the same time of the interview the previous day, up until the time of the interview), and enumerators coded the reported foods into a grid of 30 food groups, adapted from that used by the Demographic and Health Surveys (MEASURE DHS/ICF International, 2011). In addition to these 30 food groups, caregivers were asked to specifically report if their child ate the study food in the previous 24 h; as the study foods fit into categories already encompassed by the 30 food groups on the survey, enumerators were instructed not to double‐count the study food, but rather to include it as its own separate category.

Nonparticipant observations were conducted by female observers for four consecutive days in each observed household, for 12 h per observation day. Observers used a precoded grid in 30‐min time increments from 06:00 to 18:00 to record feeding and hygiene practices linked with supplementary product use, as well as the exact times of each breastfeeding occurrence. Observers were instructed to follow the children (usually this also meant the caregiver) wherever they went during the observation period, even if this meant going outside the home. The observer would start a timer any time the child was put to the caregiver's breast and stop the timer once the child was removed from the breast. Caregivers participating in observations were administered their in‐depth interviews the morning after the last full day of observation, so the interview responses and 24‐h recalls for these respondents pertain to the last day in which the observer was present in the household. All study children were breastfed during the observation period (minimum breastfeeding time among all participants is 15.5 min).

### 2.3 Variable specification

Outcomes: Relevant food groups from the 30‐item questionnaire were recoded into the seven categories of the WHO indicator for infant and young child minimum dietary diversity score (IYCMDD): grains, roots and tubers; legumes and nuts; dairy products (milk, yogurt, cheese); flesh foods (meat, fish, poultry, liver/organ meats and insects); eggs; vitamin A‐rich fruits and vegetables and other fruits and vegetables (Table 1) (World Health Organization, 2010). Note that insects are included in the flesh foods category of the indicator in this context, on the advice of nutrition experts in Burkina Faso, despite not being officially included in the WHO indicator. Regardless, insects were consumed by a small fraction of the population (19 people [1.2%]), and their inclusion does not likely influence the results.

Covariates: Child age was categorized based on the WHO recommendation that the IYCMDD indicator be disaggregated and reported for age groups: 6–11, 12–17 and 18–23 months. Though children were enrolled in the blanket supplementary feeding trial study at 6 months, the first interviews were conducted when children had been enrolled at least 1 month, so the youngest children in this subsample are 7 months old. The Household Food Insecurity Access Scale (HFIAS) (Coates, 2007) was used to create a categorical variable for food insecurity, and wealth quintiles were made using a socio‐economic status score derived from principal components analysis of household possessions. The variable for total illnesses reported over the study period adds all illnesses reported in the 2 weeks prior to the standard monthly anthropometric measurement visits. A binary variable was coded to indicate whether a family was agro‐pastoral; agro‐pastoralists make up a small percentage of the overall sample, but the distribution among the study arms is important to investigate, as there may be differing dietary practices among agro‐pastoralists (Turner, 2004) who often have more access to dairy products than those who primarily depend only on agriculture. In addition, variables for child sex, twin status, maternal age and rainy season were investigated as covariates and checked for balance among the study arms.

### 2.4 Analysis methods

We built several models to test our hypotheses about displacement of household staple cereals, nutrient‐dense foods or breastmilk as a result of supplementation with the four different supplementary foods, focusing on the differences between the three FBFs and the LNS. Separate models were built using dichotomous outcome variables indicating consumption of each of the seven food groups that comprise the IYCMDD. In addition, a composite score for dietary diversity was modelled as a continuous outcome. To investigate breastfeeding displacement, the main outcome was breastfeeding time during the 24‐h recall period in which dietary data were collected (breastfeeding time on Day 4 of the in‐home observation).

For each of the nine study outcomes, we looked at both intention to treat models where the main independent variable was assignment to study arm, and average treatment effect models in which only children who had reportedly consumed the ration during the 24‐h recall period for dietary data collection were included. Logistic regression models were estimated for dichotomous outcomes using maximum likelihood estimation procedures, and ordinary least squares linear regression models were used for continuous outcomes. The distribution of continuous variables was assessed for normality using measures of skewness and kurtosis, and if the variables were found to be skewed, models were assessed both with and without extreme outliers, as robustness checks. Model selection was done using a forward selection method, starting with no covariates and adding them in one by one until the metrics indicating model fit (*R* ^2^, residual sum of squares) no longer improved. In general, variables were also kept in the model if the point estimates for study arm changed by more than 10% with their addition; however, age was kept in the models regardless, due to its biological importance.

Covariates (Table 2) to include in modelling procedures were chosen a priori based on a conceptual framework created from literature review of similar studies (Campbell et al., 2018; Christian et al., 2015). Once linear terms were selected, interaction effects between study arm and child age, wealth status and food security were tested using likelihood ratio tests. All models were assessed for collinearity using variance inflation factor cut‐offs of 10. Correct functional form was assessed using the Ramsey RESET test. Linear regression models were assessed for heteroskedasticity and adjusted accordingly using robust standard errors. For all models, the LNS (RUSF) arm acts as the reference. All analyses were performed using Stata version 13.1 (StataCorp LP, 2013).

**TABLE 2:** Characteristics of children participating in blanket supplementary feeding program by study arm, Sanmatenga Province, Burkina Faso, 2014–2016

	Overall	CSB+ w/oil	CSWB w/oil	SC+	RUSF	*p* value^a^
**Maximum interview *n***	1,591	422	394	371	404	
**Maximum direct observation *n***	176	43	46	41	46	
Ration consumption in 24 h prior to interview	916 (59)	250 (61)	172 (45)	212 (58)	282 (72)	0.00
**Outcomes**						
Diet diversity score	2.6 ± 1.4	2.4 ± 1.4	2.6 ± 1.4	2.6 ± 1.4	2.7 ± 1.3	0.02
Grains, roots and tubers	1,344 (86)	342 (83)	328 (86)	320 (87)	354 (90)	0.04
Legumes and nuts	593 (38)	131 (32)	153 (40)	144 (39)	165 (42)	0.02
Dairy	81 (5)	9 (2)	28 (7)	19 (5)	25 (6)	0.01
Flesh foods	356 (23)	85 (21)	95 (25)	86 (23)	90 (23)	0.54
Eggs	30 (2)	7 (2)	8 (2)	6 (2)	9 (2)	0.89
Vitamin A‐rich fruits and vegetables	933 (60)	235 (57)	235 (62)	214 (58)	249 (63)	0.24
Other fruits and vegetables	670 (43)	178 (43)	151 (40)	173 (47)	168 (43)	0.23
Breastfeeding time	52.8 ± 27.4	51.5 ± 21.7	53.7 ± 30.9	51.8 ± 30.9	54.0 ± 25.7	0.95
**Covariates**						
Child age						0.62
7–11 mo	436 (27)	124 (29)	102 (26)	105 (28)	105 (26)	
12–17 mo	607 (38)	154 (37)	163 (41)	131 (35)	159 (39)	
18–23 mo	548 (34)	144 (34)	129 (33)	135 (36)	140 (35)	
Child sex						0.12
Female	800 (50)	201 (48)	197 (50)	206 (56)	196 (49)	
Male	791 (50)	221 (52)	197 (50)	165 (44)	208 (51)	
Child is a twin						0.09
Yes	57 (4)	11 (3)	15 (4)	9 (2)	22 (5)	
No	1,534 (96)	411 (97)	379 (96)	362 (98)	382 (95)	
Food security						0.11
Food secure	679 (43)	171 (41)	178 (46)	146 (40)	184 (46)	
Mildly food insecure	255 (16)	71 (17)	64 (17)	52 (14)	68 (17)	
Moderately food insecure	395 (25)	105 (25)	102 (26)	101 (28)	87 (22)	
Severely food insecure	234 (15)	68 (16)	41 (11)	63 (17)	62 (15)	
Number children <5 in HH	2.9 ± 1.9	3.1 ± 2.1	3.0 ± 2.1	2.5 ± 1.5	3.2 ± 1.9	0.00
Season						0.80
Rainy (June–September)	494 (31)	135 (32)	120 (30)	120 (32)	119 (29)	
Dry (October–May)	1,097 (69)	287 (68)	274 (70)	251 (68)	285 (71)	
Total illnesses reported over study period^b^	6.4 ± 3.4	6.3 ± 3.5	5.9 ± 3.3	6.9 ± 3.3	6.7 ± 3.5	0.00
Wealth Quintiles^c^						0.00
Lowest	319 (20)	99 (24)	61 (16)	79 (22)	80 (20)	
Mid‐low	322 (20)	83 (20)	87 (22)	78 (21)	74 (19)	
Medium	306 (20)	103 (25)	82 (21)	60 (16)	61 (15)	
Mid‐high	315 (20)	70 (17)	82 (21)	70 (19)	93 (23)	
Highest	303 (20)	57 (14)	80 (20)	78 (21)	88 (22)	
Agro‐pastoralist family						0.10
Yes	117 (7)	22 (5)	35 (9)	34 (9)	26 (6)	
No	1,474 (93)	400 (95)	359 (91)	337 (91)	378 (94)	
Maternal age						0.30
15–19	232 (16)	61 (17)	60 (17)	47 (14)	64 (18)	
20–24	381 (27)	98 (27)	98 (28)	80 (24)	105 (29)	
25–29	349 (25)	97 (27)	85 (24)	78 (23)	89 (25)	
30–34	264 (19)	64 (18)	69 (19)	75 (22)	56 (16)	
35+	189 (13)	45 (12)	42 (12)	58 (17)	44 (12)	

*Note*: Values are n (%) or mean ± SD.

Abbreviations: CSB+, Corn Soy Blend Plus; CSWB, Corn Soy Whey Blend; RUSF, Ready‐to‐use Supplementary Food; SC+, SuperCereal Plus.

^a^ *p* values for binary variables obtained using bivariate logistic regressions, *p* values for multilevel categorical variables obtained using bivariate multinomial logistic regressions, with robust SEs.

^b^ Includes the number of times fever, diarrhoea, respiratory symptoms, confirmed malaria or other illness reported in the 2 weeks prior to anthropometric measurements, throughout the study period.

^c^ Derived from principle components analysis of household materials and possessions.

### 2.5 Ethical Considerations

The study was approved by the ethics committee of the Ministry of Health of Burkina Faso, as well as the Tufts University Institutional Review Board. It is registered on clinicaltrials.gov under identifier: NCT02071563. The datasets generated and analysed during the current study will be made available on the Development Data Library of USAID, found at https://data.usaid.gov. The study protocol is available upon request.

## 3 RESULTS

### 3.1 Characteristics of sample

Characteristics of the sample are shown in Table 2. A total of 1,591 interviews and 176 in‐home observations were completed. Overall, 59% of the study subjects reported consuming the ration in the 24 h prior to the interview, though this differed significantly by study arm, with those in the RUSF arm reporting the highest level of ration consumption (72%) and those in the CSWB arm the lowest (45%). In general, the study arms are well‐balanced in terms of child and household demographic characteristics.

### 3.2 Consumption patterns

Overall, the most commonly consumed food group was grains, roots and tubers, with 86% of the overall sample reporting consumption during the recall period (Table 2). Least commonly consumed were eggs (2% overall) and dairy products (5% overall). The proportion of the sample consuming grains, roots and tubers; legumes and nuts and dairy differed significantly by study arm, with the highest proportion of those in the RUSF arm consuming grains, roots and tubers (90%) and legumes and nuts (42%), and the highest proportion of those in the CSWB w/oil arm consuming dairy (7%). There were no significant differences in consumption of the four remaining food groups across the study arms. The mean dietary diversity score, measured on a scale from 0 to 7, was 2.6 +/−1.4 overall, and varied by study arm, with the highest dietary diversity scores seen among those assigned to the RUSF arm (2.7 +/−1.3). Mean breastfeeding time in the 24 h prior to the interview was 52.8 +/−27.4 min, with those assigned to the RUSF arm clocking the longest average breastfeeding times at 54.0 +/−25.7 min. Dietary diversity is normally distributed (skewness = −0.05, kurtosis = 2.5), and after removal of four extreme outliers (breastfeeding time >120 min), breastfeeding time is also normally distributed (skewness = 0.65, kurtosis = 2.9). Breastfeeding time models were assessed both with and without these four extreme outliers and showed consistent results, thus models presented for breastfeeding time include the four outliers.

**FIGURE 1:**
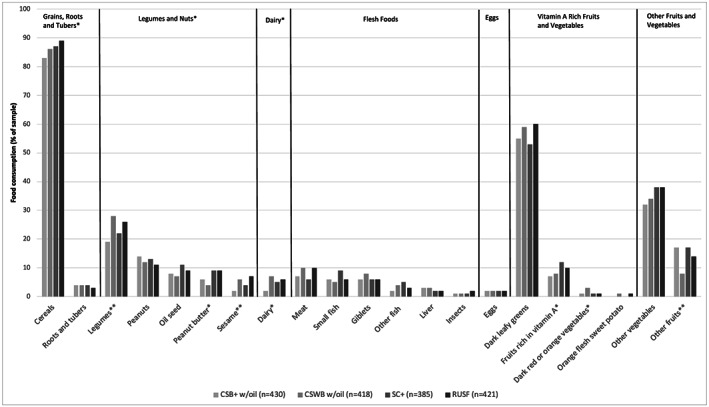
Caregiver report of household food group consumption by supplementary food type among children 7–23 months participating in a blanket supplementary feeding program, Burkina Faso 2014–2016. CSB+ = Corn Soy Blend Plus; CSWB = Corn Soy Whey Blend; SC+ = Super Cereal Plus; RUSF = Ready‐to‐use Supplementary Food. ^*^ *p* < 0.05, ^**^ *p* < 0.01, ^***^ *p* < 0.001

Figure 1 further breaks down consumption by showing each of the food subcategories that make up the IYCMDD, by study arm. While the most commonly consumed food group was grains, roots and tubers, only 4% of the sample consumed roots and tubers during the 24‐h recall period, whereas 86% consumed cereals. We therefore interpret any patterns pertaining to this food group as being driven by cereal consumption rather than consumption of roots and tubers. This is important because, while grains, roots and tubers are grouped together due to their nutritional qualities, household unfortified cereals bear a close physical resemblance to the FBFs distributed as supplements. The largest contributor to vitamin A‐rich fruits and vegetables was dark leafy greens and that of legumes and nuts was legumes, followed by peanuts. Other vegetables were more commonly consumed than other fruits.

### 3.3 Displacement of complementary food groups

**FIGURE 2:**
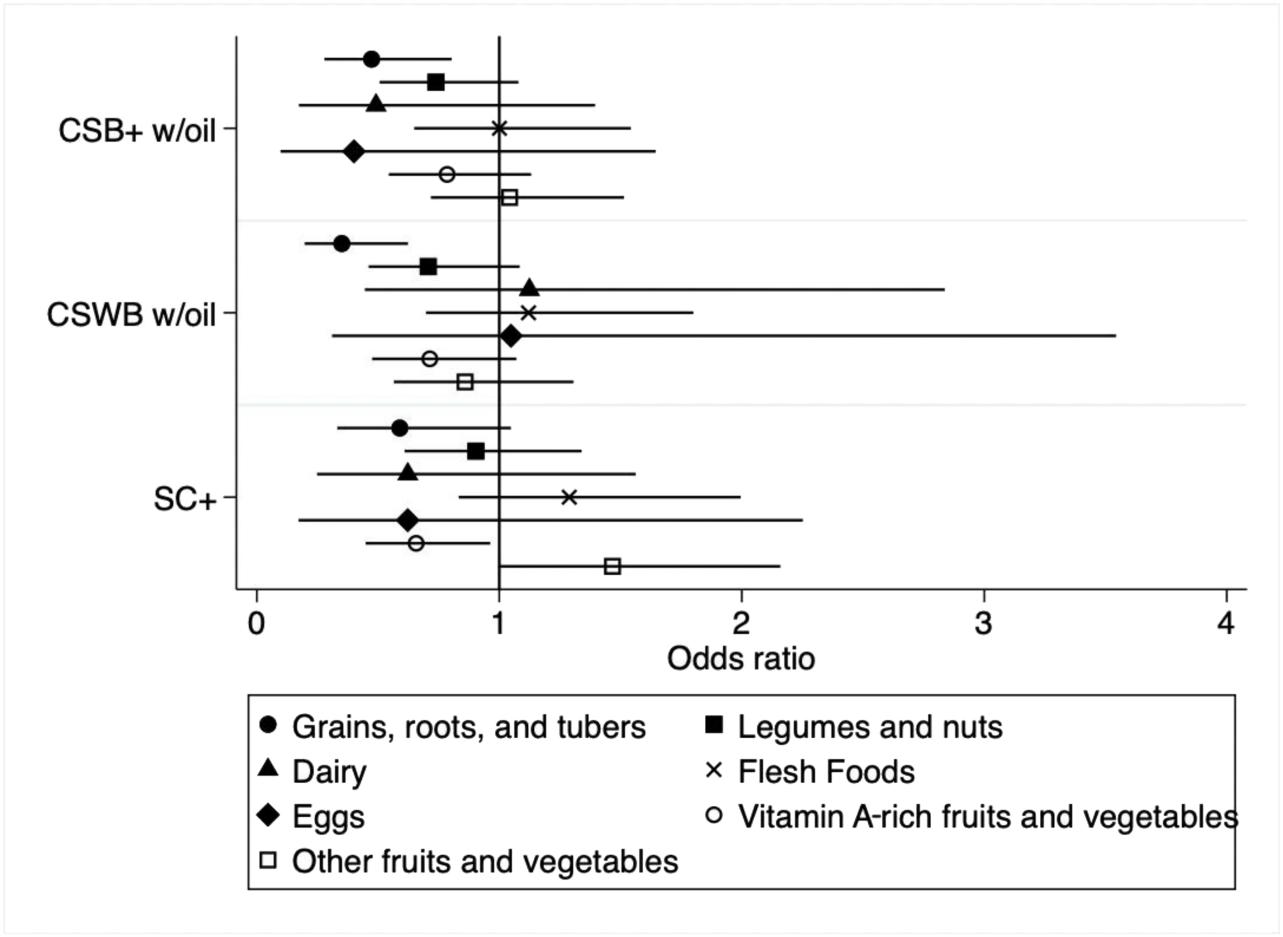
Odds ratios and 95% confidence intervals for displacement of household food groups by consumption of different supplementary food types among children 7–23 months in Burkina Faso, 2014–2016. Models adjust for child sex, age (category), total illness (2 weeks prior to each anthropometric visit) reported over study period, twin status; household food security, wealth quintile, number children <5

Comparisons of model coefficients from the average treatment effect models for each food group are displayed in Figure 2, with more details shown in Table 3. None of the interaction terms tested was significant (likelihood ratio test *p* values all above 0.1); thus, all model results are presented without interaction terms. The only food groups for which there were significant differences in consumption prevalence among study arms were grains, roots and tubers; vitamin A‐rich fruits and vegetables and other fruits and vegetables. Results from both intention‐to‐treat and average treatment effect models are consistent in showing that consumption of the grains, roots and tubers food group, which is almost entirely made up of cereals in this sample, was significantly lower among ration consumers in the FBF arms (CSB+, CSWB, SC+) than those in the LNS (RUSF) arm. The average treatment effect models showed significantly lower odds of grains, roots and tuber consumption among those that consumed CSB+ w/oil (odds ratio [OR] = 0.47; 95% confidence interval [CI]: 0.28, 0.81) and CSWB w/oil (OR = 0.35; 95% CI: 0.2, 0.62) and borderline lower odds (*p* < 0.1) among consumers of SC+ (OR = 0.59; 95% CI: 0.33, 1.05) compared with consumers of RUSF. Consumers of SC+ also had lower odds of consuming vitamin A‐rich fruits and vegetables (OR = 0.66; 95% CI: 0.45, 0.96), but borderline higher odds of consuming other fruits and vegetables (OR = 1.47; 95% CI: 1.00, 2.16) compared with RUSF consumers.

**TABLE 3:** Odds ratios from logistic regression models for consumption of household food groups and coefficients from linear regression models for dietary diversity and breastfeeding time among children 7–23 months in Burkina Faso, 2014–2016

	Crude OR	95% CI	Adjusted^a^ OR	95% CI
**Grains, roots and tubers**				
**Model 1, intention‐to‐treat (ref = RUSF)**	*n* = 1,557		*n* = 1,509	
CSB+	0.54^***^	0.36, 0.82	0.60^**^	0.38, 0.94
CSWB	0.69^*^	0.44, 1.06	0.62^**^	0.39, 0.99
SC+	0.75	0.48, 1.18	0.76	0.47, 1.22
**Model 2, average treatment effect (ref = RUSF)**	*n* = 916		*n* = 886	
CSB+	0.42^****^	0.26, 0.67	0.47^***^	0.28, 0.81
CSWB	0.42^**^	0.25, 0.71	0.35^****^	0.20, 0.62
SC+	0.65	0.38, 1.09	0.59^*^	0.33, 1.05
**Vitamin A‐rich fruits and vegetables**				
**Model 1**	*n* = 1,557		*n* = 1,509	
CSB+	0.77	0.58, 1.02	0.83	0.62, 1.12
CSWB	0.93	0.70, 1.24	0.94	0.70, 1.28
SC+	0.81	0.60, 1.08	0.82	0.60, 1.11
**Model 2**	*n* = 916		*n* = 886	
CSB+	0.73^*^	0.52, 1.03	0.78	0.54, 1.13
CSWB	0.72^*^	0.49, 1.05	0.71	0.48, 1.07
SC+	0.66^**^	0.46, 0.95	0.66^**^	0.45, 0.96
**Other fruits and vegetables**				
**Model 1**	*n* = 1,557		*n* = 1,509	
CSB+	1.02	0.77, 1.35	1.11	0.83, 1.49
CSWB	0.88	0.66, 1.17	0.88	0.65, 1.19
SC+	1.19	0.89, 1.59	1.26	0.93, 1.70
**Model 2**	*n* = 916		*n* = 886	
CSB+	0.91	0.64, 1.29	1.04	0.72, 1.51
CSWB	0.80	0.54, 1.19	0.86	0.57, 1.31
SC+	1.31	0.92, 1.88	1.47^*^	1.00, 2.16
**Legumes and nuts**				
**Model 1**	*n* = 1,557		*n* = 1,509	
CSB+	0.64^***^	0.48, 0.86	0.71^**^	0.52, 0.96
CSWB	0.93	0.70, 1.23	0.94	0.70, 1.27
SC+	0.89	0.67, 1.19	0.96	0.70, 1.30
**Model 2**	*n* = 916		*n* = 886	
CSB+	0.63^**^	0.44, 0.90	0.74	0.51, 1.08
CSWB	0.75	0.51, 1.11	0.71	0.46, 1.08
SC+	0.86	0.60, 1.24	0.90	0.61, 1.34
**Dairy** ^b^				
**Model 1**	*n* = 1,556		*n* = 1,454	
CSB+	0.33^***^	0.15, 0.71	0.36^**^	0.15, 0.84
CSWB	1.17	0.67, 2.04	1.20	0.65, 2.26
SC+	0.80	0.43, 1.49	0.57	0.28, 1.19
**Model 2**	*n* = 916		*n* = 855	
CSB+	0.38^**^	0.15, 0.99	0.49	0.17, 1.40
CSWB	1.07	0.48, 2.29	1.12	0.44, 2.83
SC+	0.85	0.39, 1.86	0.62	0.25, 1.56
**Flesh foods**				
**Model 1**	*n* = 1,557		*n* = 1,509	
CSB+	0.87	0.63, 1.22	0.98	0.69, 1.39
CSWB	1.12	0.80, 1.56	1.19	0.84, 1.68
SC+	1.03	0.74, 1.44	1.12	0.78, 1.59
**Model 2**	*n* = 916		*n* = 886	
CSB+	0.91	0.60, 1.38	1.00	0.65, 1.54
CSWB	1.09	0.69, 1.70	1.12	0.70, 1.80
SC+	1.19	0.78, 1.80	1.29	0.83, 2.00
**Eggs**				
**Model 1**	*n* = 1,557		*n* = 1,509	
CSB+	0.74	0.27, 2.00	0.67	0.24, 1.86
CSWB	0.92	0.34, 2.40	0.74	0.27, 2.05
SC+	0.71	0.25, 2.01	0.62	0.21, 1.79
**Model 2**	*n* = 916		*n* = 886	
CSB+	0.48	0.12, 1.87	0.40	0.10, 1.64
CSWB	1.42	0.47, 4.30	1.05	0.31, 3.54
SC+	0.76	0.22, 2.61	0.62	0.17, 2.25
	Crude 𝛽	95% CI	Adjusted^a^ 𝛽	95% CI
**Dietary diversity score**				
**Model 1**	*n* = 1,557		*n* = 1,509	
CSB+	−0.31^***^	−0.49, −0.11	−0.19^**^	−0.38, −0.00
CSWB	−0.08	−0.28, 0.12	−0.06	−0.25, 0.13
SC+	−0.08	−0.28, 0.12	−0.03	−0.22, 0.16
**Model 2**	*n* = 916		*n* = 886	
CSB+	−0.39^***^	−0.64, −0.14	−0.24^*^	−0.48, 0.00
CSWB	−0.29^**^	−0.57, −0.01	−0.28^**^	−0.55, −0.01
SC+	−0.10	−0.36, 0.16	−0.05	−0.32, 0.19
**Breastfeeding time, Day 4 of observation**				
**Model 1**	*n* = 191		*n* = 182	
CSB+	−2.51	−13.61, 8.58	−2.34	−13.34, 8.66
CSWB	−0.32	−11.24, 10.60	−7.65	−18.79, 3.49
SC+	−2.16	−13.38, 9.06	−0.35	−11.61, 10.92
**Model 2**	*n* = 91		*n* = 86	
CSB+	−3.84	−19.89, 12.21	−8.01	−22.91, 6.89
CSWB	−0.05	−15.59, 15.49	−13.61^**^	−29.06, 1.84
SC+	−9.44	−25.90, 7.02	−7.45	−23.25, 8.33

Abbreviations: CI, confidence interval; CSB+, Corn Soy Blend Plus; CSWB, Corn Soy Whey Blend; OR, odds ratio; RUSF, Ready‐to‐use Supplementary Food; SC+, SuperCereal Plus.

^a^ Adjusted models control for: child characteristics; sex, age category, total illnesses (2 weeks prior to each anthropometric visit) reported over study period, twin status, Household factors; food security (categorical), wealth (quintiles), number children <5.

^b^ Dairy models additionally control for agro‐pastoralist family due to significance of agro‐pastoralism to dairy consumption.

* *p* < 0.1.

** *p* < 0.05.

*** *p* < 0.01.

**** *p* < 0.001.

Adjusted models for the four remaining food groups (legumes and nuts, dairy, flesh foods and eggs) mostly show null results. Intention‐to‐treat models show lower odds of consuming both legumes and nuts (OR = 0.71; 95% CI: 0.52, 0.96) and dairy (OR = 0.36; 95% CI: 0.15, 0.84) among those assigned to the CSB+ w/oil arm; however, these significant effects are not seen in the average treatment effect models among actual consumers of CSB+ w/oil. In addition, results for both dairy and eggs should be interpreted with caution due to the homogeneity in the outcome; only 2%–7% of study participants consumed items from these food groups, depending on the study arm.

### 3.4 Dietary diversity

Results from linear regressions modelling the effects of supplement type on an overall dietary diversity score are presented in Table 3. Consumers of CSB+ w/oil and CSWB w/oil had dietary diversity scores 0.24 (95% CI: −0.48, 0.00) and 0.28 (95% CI: −0.55, 0.01) points below consumers of RUSF, respectively, representing an 8% and 10% decrease from the mean dietary diversity score of 2.9, though these results are not statistically significant at the 5% alpha level, and the CIs include the null value.

### 3.5 Breastfeeding

Table 3 also presents the results from linear regression models estimating the effects of the different supplement types on average breastfeeding time during a portion of the 24‐h recall period. No significant differences in breastfeeding time were observed for consumers of CSB+ w/oil or SC+, compared with RUSF consumers. Children who consumed CSWB w/oil during the recall period breastfed for 13.61 min less time (95% CI: −29.06, 1.84) than those who consumed RUSF (*p* < 0.1), representing a 26% shorter breastfeeding time than the average time of 52.9 min; however, this should be interpreted with caution as the result is not significant at the 5% level, and the CI crosses the null value and is quite large due to the small sample size for these models.

## 4 DISCUSSION

In this reanalysis of data from a supplementary feeding cost‐effectiveness trial, we found that supplementation with different FBFs appears to displace household cereal consumption significantly when compared to supplementation with an LNS. The relationship is strongest for two of the three FBFs tested, CSB+ w/oil and CSWB w/oil. While there is some evidence that the third FBF tested, SC+, may also displace more vitamin A‐rich fruits and vegetables than the LNS product tested, children who consumed SC+ were also more likely to eat other fruits and vegetables, indicating that those who consumed SC+ consumed similar amounts of fruits and vegetables as those in the other study arms, but consumed different types. None of the remaining food groups that make up the IYCMDD showed compelling evidence of differential displacement by the FBFs compared with the LNS product (RUSF).

Our results add nuance to previous literature showing that FBFs displace more complementary foods than LNS by offering insight into which food groups are being displaced (Flax et al., 2015; Ickes et al., 2015; Lin et al., 2008; Maleta et al., 2004; Thakwalakwa et al., 2015). Maleta et al. (2004) found that children who received maize and soy flours as food supplements consumed less of household staple foods than those who received an LNS. In a separate study, Thakwalakwa et al. (2015) found that mean total energy intake in an LNS group was greater than in a CSB group and that there was less displacement of non‐supplement foods with LNS compared with CSB, as in our study.

Although the intention of both FBFs and LNS is that they serve as supplements and not replacements for household foods, the displacement of household cereals but not other, more nutrient‐dense foods by FBFs may assuage some worries that supplementary foods may have limited effectiveness due to displacement of household foods. Given the small stomach size and limited feeding time of infants (Dewey, 2013), this displacement of unfortified household cereals by fortified flours may be beneficial for infants to meet their nutrient needs. The FBFs are providing an enhanced nutrient profile compared with household staple cereals, so consumption of these more nutrient‐dense cereals in place of household staple cereals could be beneficial. Results of our main cost‐effectiveness trial showing that the CSB+ w/oil was the most cost‐effective food among those tested in preventing stunting and wasting (Cliffer et al., 2020) support this claim; perhaps displacement of household cereals by the CSB+ w/oil (and the other FBFs) is one mechanism by which its comparable effectiveness and thus superior cost‐effectiveness to RUSF in preventing stunting and wasting is achieved.

These results are consistent with our hypotheses predicting that FBFs would replace household root crops and cereal grains due to their close physical resemblance to each other (Sawadogo et al., 2010), but not replace other, more nutrient‐dense foods that lack the resemblance. Fortified flour closely resembles traditional flours made from household cereals, is prepared in a similar manner and is consumed the same way (Sawadogo et al., 2010). This similarity could explain why FBFs are more likely to displace cereal than LNS products. Caregiver time is valuable, and the preparation time and process required for the use of both household cereals and FBFs may keep people from preparing similar foods multiple times a day (Maleta et al., 2004; Shen et al., 2020). In contrast, feeding a ready‐to‐use LNS requires little additional time from the caregiver (Shen et al., 2020), and thus would not need to replace any other food groups, because there is little lost opportunity cost to make up for. The slight differences seen in the behaviour of the SC+ consumers compared with the other two FBFs supports this theory; the SC+ bears less of a physical resemblance to normal household cereals than do the other two FBFs, as it is a lighter colour, tastes sweeter and is finer than the other cereals (Saleh, 2013) and is thus often served to the children raw instead of being cooked into a porridge. Therefore, the finding that consumers of SC+ had only slightly lower odds of eating grains, roots and tubers (mostly cereals) compared with consumers of RUSF, whereas the relationships were stronger for CSB+ w/oil and CSWB w/oil may be due to the fact that SC+ is perceived as a more novel cereal than the other two FBFs.

In addition, if the perception is that the supplementary food is giving the child an enhanced nutrient profile, as was reported by 20% of caregivers in our sample when asked why their children received the food supplements, and children have limited capacity for caloric intake, caregivers may prefer to substitute the FBFs for their inferior household grains but may perceive the LNS as an extra food because it does not closely resemble any existing household foods. While we lack data on quantities of foods consumed and can therefore not conclude anything about specific nutrient consumption, these findings still add valuable insight to our understanding of caregiver complementary feeding behaviours in the presence of different types of food supplements.

The borderline significant reductions (at the 10% significance level) in dietary diversity that we observed in conjunction with consumption of CSB+ w/oil and CSWB w/oil may also be due to the exclusion of household cereals from the diets of children who consume those FBFs and the replacement of these cereals with the FBFs. Current literature by Campbell et al. (2016) demonstrates that the provision of supplementary foods may increase dietary diversity. Unfortunately, a major limitation of our study is the lack of a pure control group with which to compare, so we can draw no conclusions about absolute changes in dietary diversity due to supplementary feeding.

Though our sample size is quite small for breastfeeding observations, and we only have data on breastfeeding times during the 12‐h observation period from 6:00–18:00, which limits interpretation of these results, our results show borderline significant reductions in breastfeeding time among consumers of CSWB w/oil and nonstatistically significant reductions in breastfeeding time among consumers of the other two FBFs compared with the LNS products. As with interpretation of the dietary diversity score results, we are limited to the comparison of FBFs to the LNS product and cannot draw any conclusions about absolute displacement of breastmilk by food supplements compared to a pure control group. These results, however, align with previous literature showing no significant displacement of breastmilk by LNS foods (Campbell et al., 2016; Galpin et al., 2007; Kumwenda et al., 2014; Owino et al., 2007). Though we find no studies that show the relationship between breastfeeding time and nutrient consumption, because higher breastfeeding frequency is associated with greater milk intake, more nutrients and better growth (Gridneva et al., 2018), the finding that consumers of the FBFs breastfed for shorter amounts of time than consumers of the LNS product could mean that consumers of FBFs obtain less breastmilk, but this result must be interpreted with caution and is not statistically significant at the 5% alpha level.

## 5 CONCLUSIONS

In the context of a blanket supplementary feeding program designed to prevent undernutrition in young children in Burkina Faso, it appears that FBFs have significantly higher odds of displacing household grains, roots and tubers (mainly cereals) than does the LNS product. This could have implications in terms of the effectiveness of supplementary feeding programs. While both the current and previous studies have shown that FBFs displace more household foods than LNS, it is reassuring that the only household foods that are being displaced appear to be household cereals which are likely inferior in nutritional quality to the FBFs that replace them. This provides a likely mechanism by which FBFs can be as effective as LNS products in preventing stunting and wasting, as was demonstrated by the primary results of the cost‐effectiveness trial from which these secondary analyses were derived (Cliffer et al., 2020).

While our study was limited in that we were not able to calculate specific quantities of foods consumed, but rather used dichotomous indicators of consumption of the food groups, nor were we able to compare with a pure control group, the results are helpful to supplementary feeding programmers who need to decide which products to program for prevention and treatment of undernutrition. Future studies should investigate any potential consequences of displacement of household cereals and estimate the quantities of each food group that could be displaced by supplementary foods.

## CONFLICTS OF INTEREST

None of the authors report conflicts of interest.

## CONTRIBUTIONS

BR designed original study; IC and WM designed re‐analysis. IC conducted research, analysed data, wrote the paper. WM and BR critically reviewed the paper. IC had primary responsibility for final content. All authors have read and approved the final manuscript.
